# Metabolomics profiling identifies diagnostic metabolic signatures for pregnancy loss: a cross-sectional study from northwestern China

**DOI:** 10.3389/fendo.2025.1518043

**Published:** 2025-04-10

**Authors:** Nan Ding, Xin Yang, Ruifang Wang, Fang Wang

**Affiliations:** Reproductive Medicine Center, Lanzhou University Second Hospital, Lanzhou, China

**Keywords:** pregnancy loss, untargeted metabolomics, LASSO regression, metabolic signature, diagnosis

## Abstract

**Objective:**

To identify potential diagnostic metabolic biomarkers for pregnancy loss (PL) by performing untargeted metabolomics analysis.

**Methods:**

The present study performed untargeted metabolomics analysis on plasma samples from PL patients (n=70) and control subjects (n=122) using liquid chromatography‒mass spectrometry (LC‒MS). Metabolic profiles were evaluated using orthogonal partial least squares discriminant analysis (OPLS-DA), and pathway enrichment analysis was conducted via the KEGG database. LASSO regression was employed to identify significant metabolites, and their diagnostic performance was evaluated through receiver operating characteristic (ROC) curves. Pearson correlation analysis was used to explore the relationships between differentially abundant metabolites and clinical parameters.

**Results:**

In total, 359 metabolites were identified, 57 of which were significantly altered between the control and PL group through OPLS-DA. Differential metabolites were significantly enriched in caffeine metabolism, tryptophan metabolism, and riboflavin metabolism pathways. Key metabolites, such as testosterone glucuronide, 6-hydroxymelatonin, and (S)-leucic acid, exhibited strong diagnostic potential, with AUC values of 0.991, 0.936 and 0.952, respectively, and the combined AUC was 0.993. Furthermore, Pearson correlation analysis revealed a significant negative correlation between the waist‒to‒hip ratio (WHR) and the abundance of testosterone glucuronide (r = -0.291, p = 0.0146), and a significant positive correlation between WHR and (S)-leucic acid (r = 0.248, p = 0.0381) in the PL group.

**Conclusion:**

We identified a panel of plasma metabolites with significant diagnostic potential for PL. These biomarkers may facilitate early, noninvasive diagnosis and offer insights into metabolic dysregulation associated with pregnancy loss.

## Introduction

Pregnancy loss (PL) refers to the natural end of a pregnancy before the fetus becomes viable, including all losses from conception until 24 weeks of gestation, affecting approximately 12–15% of recognized pregnancies globally (1, 2). Owing to increased environmental pollution and increasing life stress, the clinical incidence of PL has significantly increased in recent years (3, 4). Despite extensive research, the underlying causes of PL remain poorly understood and are often attributed to a complex interplay of genetic, immunological, and environmental factors (5). The absence of reliable metabolites complicates the early diagnosis and management of PL, leading to profound psychological and physiological impacts on affected women and their families (6).

In the context of PL, metabolomics can reveal critical metabolic alterations associated with this condition (7). Previous research has shown the effectiveness of metabolomics in identifying metabolic signatures for numerous conditions, such as cancer, cardiovascular diseases, autoimmune disease and metabolic disorders (8–11). For example, Lili Zhang et al. investigated serum metabolic profiles in women with recurrent abortion due to antiphospholipid syndrome, and they reported significant disruptions in purine, amino acid, and tyrosine metabolism (12). Another study utilized untargeted GC–MS and targeted liquid chromatography–mass spectrometry (LC–MS) to identify metabolic disturbances associated with recurrent spontaneous abortion. Through this approach, lactic acid and 5-methoxytryptamine were identified as significantly different metabolites between the two groups, and their plasma concentrations were further validated using targeted LC–MS (13). Despite these advances, comprehensive metabolomics analyses specifically targeting PL remain limited.

The integration of metabolomics with well-established analytical techniques, such as LC–MS, enables the detection of subtle metabolic alterations associated with PL. Moreover, the application of machine learning algorithms to metabolomics data has further improved the ability to identify and validate potential metabolites (14). One such machine learning algorithm, least absolute shrinkage and selection operator (LASSO) regression, is particularly effective in managing high-dimensional data characteristic of complex datasets (15, 16). LASSO regression stands out for its ability to perform both variable selection and regularization simultaneously, which helps prevent overfitting while managing large datasets (17). This is especially beneficial in metabolomics, where the number of metabolites often far exceeds the number of samples. By shrinking the coefficients of less relevant variables to zero, LASSO effectively refines the model, focusing on the most significant metabolites (18). This enhances the robustness of the model and increases the reproducibility of identifying the most important metabolites.

The present study aimed to leverage untargeted metabolomics combined with LASSO regression to identify a panel of diagnostic metabolites for PL. By comparing the metabolic profiles of plasma samples from women with a history of PL to those from women who have had normal pregnancies without PL, the present study sought to uncover metabolic signatures that can aid in the early detection and better understanding of PL. In addition, analyses that incorporated clinical parameters, such as age, BMI, and waist–to–hip ratio (WHR), which are potential confounding factors in PL, were performed to explore the relationships between the identified metabolites and these variables. The present findings may provide valuable insights for developing noninvasive diagnostic metabolites for PL.

## Methods

### Study design and participants

The present study enrolled 192 participants, comprising 122 in the control group and 70 in the PL group. The participants were recruited from Lanzhou University Second Hospital between February 2023 and September 2023. The inclusion criteria for the PL group included women aged 18 to 42 years who had experienced at least one PL in the past six months, in accordance with the ESHRE diagnostic criteria (1). The exclusion criteria for the PL group were as follows: presence of endocrine disorders, infections, or immunological diseases; or a history of the most recent pregnancy ending in an ectopic pregnancy, hydatidiform mole, or congenital defects. The inclusion criteria for the control group were women with no history of PL and at least one successful full-term pregnancy. The exclusion criteria for the control group included the presence of endocrine disorders, infections, or immunological diseases. The present study was approved by the Institutional Review Board of Lanzhou University Second Hospital (ethical approval number: 2023A-553). All patients provided written informed consent.

### Sample collection and preparation

Fasting blood samples were collected from participants using EDTA tubes and centrifuged at 1,500×g for 10 minutes at 4°C to obtain plasma, which was then stored at -80°C. For metabolomics analysis, 100 μL of plasma was placed in Eppendorf tubes, mixed with 80% prechilled methanol, and vortexed thoroughly. The samples were incubated on ice for 5 minutes, followed by centrifugation at 15,000×g for 20 minutes at 4°C. A portion of the supernatant was diluted with LC–MS-grade water to reach a 53% methanol concentration. This mixture was transferred to fresh Eppendorf tubes and centrifuged again at 15,000×g for 20 minutes at 4°C. The final supernatant was then injected into the LC–MS/MS system for analysis via an Orbitrap Q Exactive™ HF–X mass spectrometer (19, 20).

### UHPLC–MS analysis

UHPLC–MS/MS analysis was performed using a Vanquish UHPLC system coupled with an Orbitrap Q Exactive™ HF-X mass spectrometer (Thermo Fisher, Germany) at Novogene Co., Ltd. Samples were injected onto a Hypersil Gold column (100 × 2.1 mm, 1.9 μm) and separated over a 12-minute linear gradient at 0.2 mL/min. The eluents for positive polarity mode consisted of 0.1% formic acid in water (eluent A) and methanol (eluent B), whereas those for negative polarity mode included 5 mM ammonium acetate (pH 9.0, eluent A) and methanol (eluent B). The solvent gradient was programmed as follows: initial 2% B for 1.5 minutes, increased to 85% B over 3 minutes, increased to 100% B over the next 10 minutes, decreased to 2% B over 0.1 minutes, and maintained at 2% B for 12 minutes. The mass spectrometer was operated in both positive and negative modes with a spray voltage of 3.5 kV, capillary temperature of 320°C, sheath gas flow rate of 35 psi, auxiliary gas flow rate of 10 L/min, S-lens RF level of 60, and auxiliary gas heater temperature of 350°C. High-energy collision dissociation (HCD) was used as the fragmentation method, with an isolation window scanning the *m/z* range of 100–1,500. Collision energy was applied in three steps, namely, 20 V, 40 V, and 60 V. The quality control (QC) samples were interspersed among the study samples during analysis. The QC samples were used to monitor and assess the data quality by evaluating the correlation between QC injections, ensuring the reliability and consistency of the analytical results. All samples were analyzed in a single batch during the analytical run.

### Data processing and metabolite identification

The raw data files from UHPLC–MS/MS were processed using Compound Discoverer 3.3 for peak alignment, peak picking, and metabolite quantitation, following the protocols established in previous studies (21, 22). First, retention times and mass-to-charge ratios (*m/z*) were aligned across samples with a mass tolerance of 5 ppm, a signal intensity tolerance of 30%, and a defined intensity threshold. Metabolite identification was performed by matching accurate *m/z* values, adduct ions, and MS/MS fragmentation patterns against the mzCloud (https://www.mzcloud.org/), mzVault, and MassList databases. In databases containing fragmentation data, the acquired MS spectra were compared with reference fragment ions and collision energies, ensuring higher confidence annotations. Next, peak intensities were normalized to reduce run-to-run variations and enable consistent comparative analyses. Quality control (QC) samples were injected to monitor instrument stability, and only features with a coefficient of variation (CV) < 30% in the QC samples were retained. Finally, the normalized peak areas of the confidently identified metabolites were used for subsequent statistical analyses.

### Data analysis

The identified metabolites were annotated using the KEGG and HMDB databases (https://www.metaboanalyst.ca/). Since our research focus is not on exogenous metabolites, we have excluded them from further analysis. A detailed list of these excluded exogenous metabolites is provided in **Supplementary Table 1**. To visualize metabolic differences and identify potential metabolic signatures, orthogonal partial least squares discriminant analysis (OPLS-DA) was performed using the ‘ropls’ package in R software. Statistical significance was assessed using the t test, with differentially abundant metabolites selected on the basis of a variable importance in projection (VIP) score > 1 and a p value < 0.05. Heatmaps and volcano plots were generated using the ‘pheatmap’ and ‘ggplot2’ packages in R. Pathway analysis was conducted using the MetaboAnalyst platform (version 6.0), a widely used web-based tool for metabolomics data interpretation. The list of significantly altered metabolites was uploaded using HMDB (Human Metabolome Database) IDs, and these metabolites were assigned on the basis of MS/MS spectral matching and database searches. The KEGG database was used for pathway enrichment analysis. Enrichment analysis was performed using the hypergeometric test to identify overrepresented pathways compared with a reference metabolome; pathways with a p value < 0.05 were considered statistically significant. To assess the biological importance of metabolites within pathways, the relative-betweenness centrality algorithm was applied during pathway topology analysis. The results were visualized via a pathway impact plot, which integrates enrichment and topology analyses to highlight the most significantly impacted pathways.

### LASSO regression for metabolites selection and ROC curve analysis

LASSO regression was employed via the ‘glmnet’ package in R to identify significant metabolites associated with PL (23). The data were divided into a training set (60% of the data) and a validation set (40% of the data). Within the training set, 10-fold cross-validation was applied using the ‘caret’ package in R to optimize the model and avoid overfitting (24). The selected features from the LASSO model were then validated using the validation set. ROC curve, sensitivity, specificity, and confusion matrix analyses were conducted using the ‘pROC’ package in R. Additionally, ROC curve analysis on clinical data, including age, BMI, and WHR, was conducted to compare their predictive performance with that of the differentially metabolite abundance-based model.

### Correlation analysis

Pearson correlation analysis was performed using the ‘Hmisc’ package in R to investigate the relationships between differentially abundant metabolites and clinical parameters, such as age, BMI, and WHR. Heatmaps were created with the ‘ComplexHeatmap’ package, and significant correlations were visualized using scatter plots generated with the ‘ggplot2’ package, offering insights into the metabolic alterations linked to clinical features in the control and PL group. Additionally, a permutation test with 200 iterations was employed to assess the statistical significance of the correlation coefficient differences between the control group and PL group.

## Results

### Baseline clinical characteristics

**Table 1** presents the baseline clinical characteristics of the study participants, which included 122 women in the control group and 70 women in the PL group. The median age in the PL group was significantly lower than that in the control group (30 years vs. 33 years, p < 0.001). The PL group had a higher median BMI (22.6 kg/m² [Q1-Q3: 21.5-24.7]) than the control group (21.8 kg/m² [Q1-Q3: 20.2-23.4], p = 0.006). Furthermore, the PL group presented a significantly greater WHR (0.9 [Q1-Q3: 0.8-0.9]) than the control group (0.8 [Q1-Q3: 0.8-0.8], p < 0.001). In terms of race, the majority of participants in both groups were Han Chinese, with no significant difference observed between the two groups (p = 0.726). However, education levels significantly differed between the two groups. A significantly greater proportion of women in the control group had a university education or above (91% vs. 67.1%, p < 0.001), whereas the PL group had a greater percentage of participants with a high school education or below (32.9% vs. 9%, p < 0.001). With respect to the number of pregnancy losses, none of the women in the control group experienced a loss, whereas the PL group included participants with varying numbers of losses, including 17.1% with one loss, 50% with two losses, and 32.9% with three or more losses (p < 0.001).

**Table 1:** Clinical characteristics of PL patients and controls.

Variables	Total (n = 192)	Control (n = 122)	PL (n = 70)	p
Age (year)	32 (29.8, 36)	33 (30.2, 37)	30 (28, 32.8)	< 0.001
BMI (kg/m2)	22 (20.6, 23.5)	21.8 (20.2, 23.4)	22.6 (21.5, 24.7)	0.006
WHR	0.8 (0.8, 0.9)	0.8 (0.8, 0.8)	0.9 (0.8, 0.9)	< 0.001
Race, n (%)				0.726
Han Chinese	183 (95.3)	117 (95.9)	66 (94.3)	
Other ethnicities	9 (4.7)	5 (4.1)	4 (5.7)	
Education, n (%)				< 0.001
High school or below	34 (17.7)	11 (9)	23 (32.9)	
University or above	158 (82.3)	111 (91)	47 (67.1)	
Number of PL, n (%)				< 0.001
0	122 (63.5)	122 (100)	0 (0)	
1	12 (6.2)	0 (0)	12 (17.1)	
2	35 (18.2)	0 (0)	35 (50)	
≥3	23 (12)	0 (0)	23 (32.9)	

BMI, Body mass index; WHR, Waist-Hip Ratio; PL, Pregnancy loss. p<0.05 was considered statistically significant.

### OPLS-DA model and permutation test

**Figure 1:**
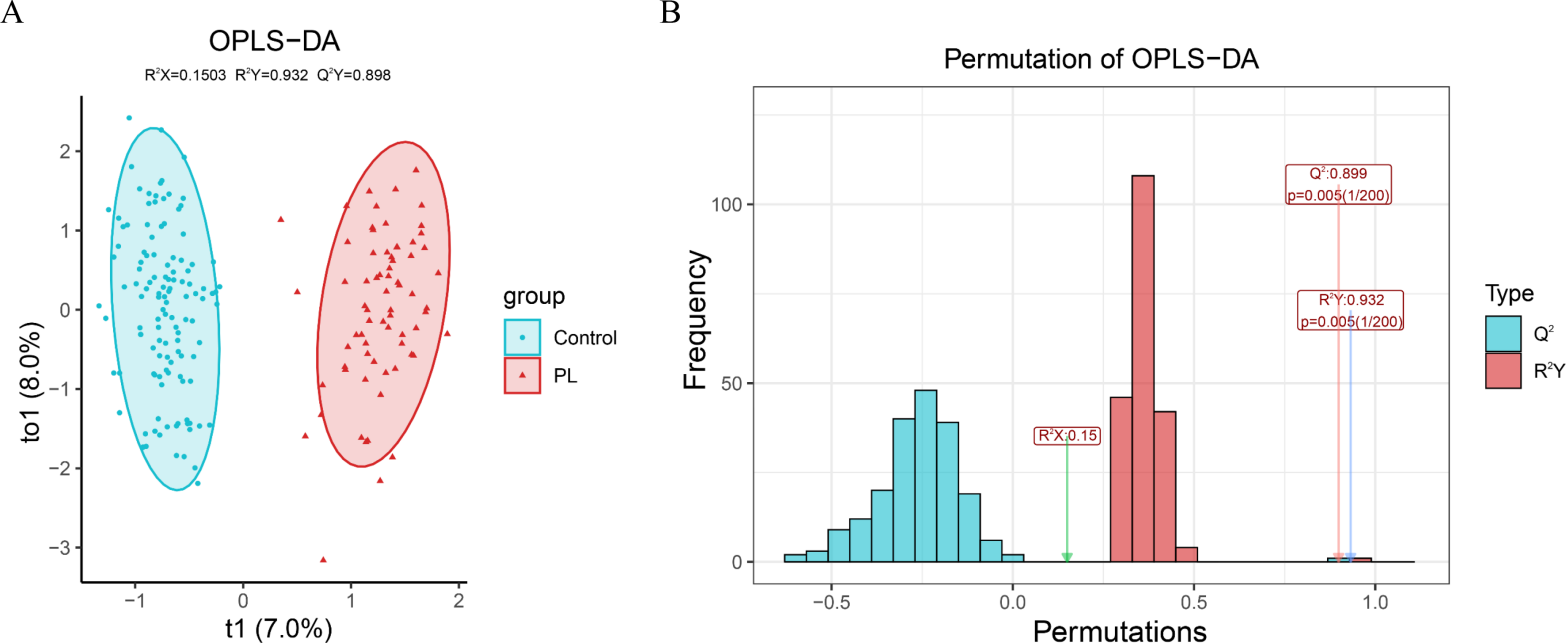
OPLS-DA model and permutation test evaluation. **(A)** OPLS-DA score plot showing the separation between the PL group and the control group based on their metabolic profiles. **(B)** Permutation test results with 200 iterations to evaluate the robustness of the OPLS-DA model.

**Figure 1** illustrates the OPLS-DA model and its evaluation through permutation tests. The OPLS-DA plot (**Figure 1A**) revealed a separation between the control and PL group, indicating distinct metabolic profiles. The x-axis (t1) explained 7.0% of the variance between the two groups, whereas the y-axis (to1) explained 8.0% of the variance within each group. A permutation test with 200 iterations (**Figure 1B**) was used to evaluate the OPLS-DA model. The histogram shows the frequency distribution of the permuted R^2^Y and Q^2^ values, with an R^2^Y value of 0.932 and an Q^2^ value of 0.899, which were both significantly higher than those obtained from the permuted models (p = 0.005), confirming the reliability and predictive performance of the model.

### Differential metabolite analysis

**Figure 2:**
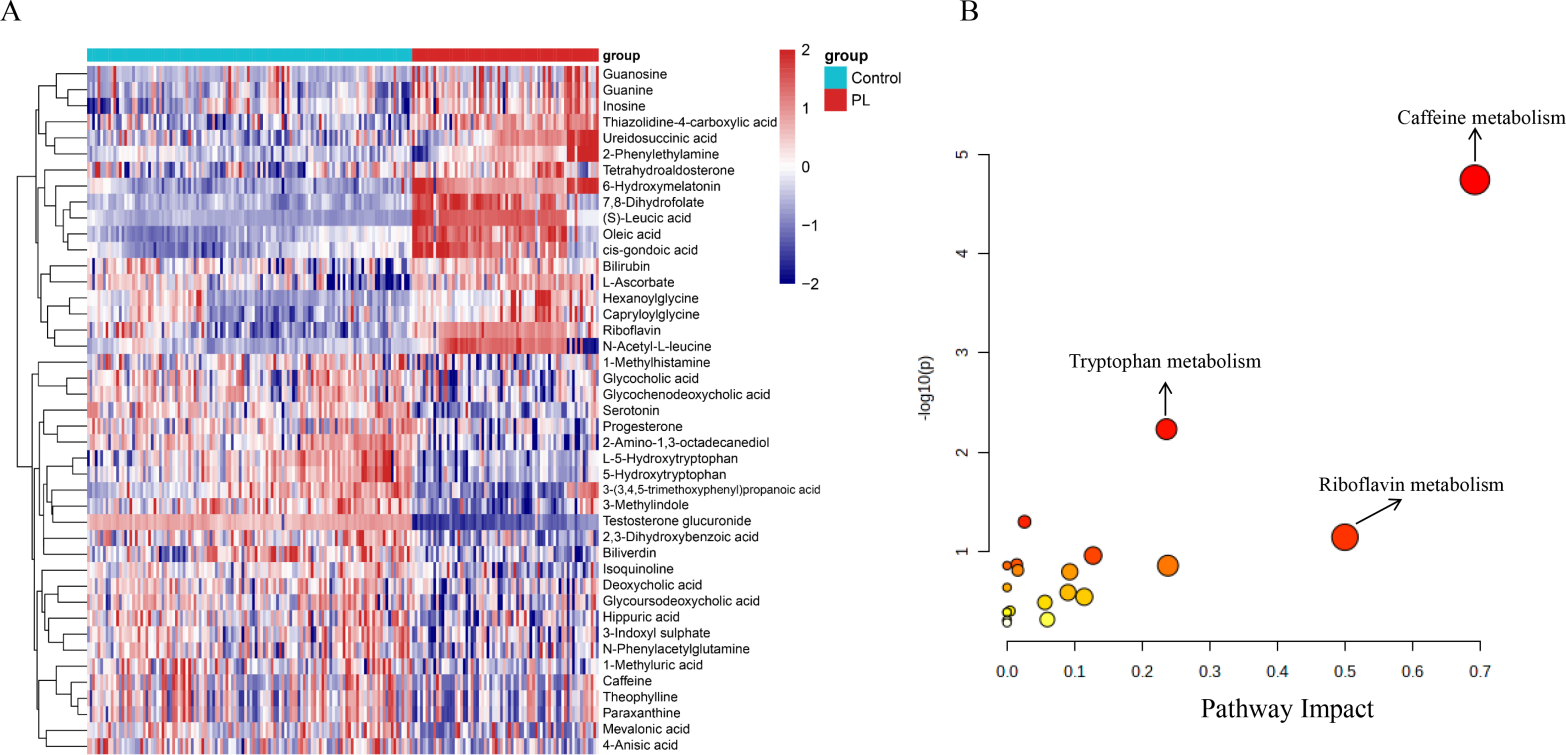
Differential metabolites and pathway enrichment analysis. **(A)** Heatmap of the top 43 differential metabolites identified between the control and PL group, showing distinct clustering patterns. **(B)** Pathway enrichment analysis bubble plot. The x-axis represents the pathway impact, and the y-axis represents the -log10 of the p-value.

**Figure 2** shows an in-depth analysis of the differentially abundant metabolites between the control and PL group. In total, 57 differentially abundant metabolites were identified on the basis of VIP> 1 and p values<0.05 (**Supplementary Table 2**). The heatmap shown in **Figure 2A** displays the differentially abundant metabolites, revealing distinct clustering patterns between the two groups. In addition, the volcano plot shown in **Supplementary Figure S1A** illustrates the differentially abundant metabolites using log2-fold changes and -log10 p values. Testosterone glucuronide, 6-hydroxymelatonin, and (S)-leucic acid demonstrated statistical significance and fold changes, with testosterone glucuronide exhibiting the greatest significance and fold change. Among the 57 significantly altered metabolites, 54 were successfully matched to known metabolic pathways in the KEGG database using the MetaboAnalyst platform. The pathway impact plot revealed several significantly impacted pathways, including caffeine metabolism, tryptophan metabolism, and riboflavin metabolism (**Figure 2B**). The bubble plot shows these pathways, with larger and more vividly colored bubbles indicating pathways with greater impact and significance.

### Screening of differential metabolites

**Figure 3:**
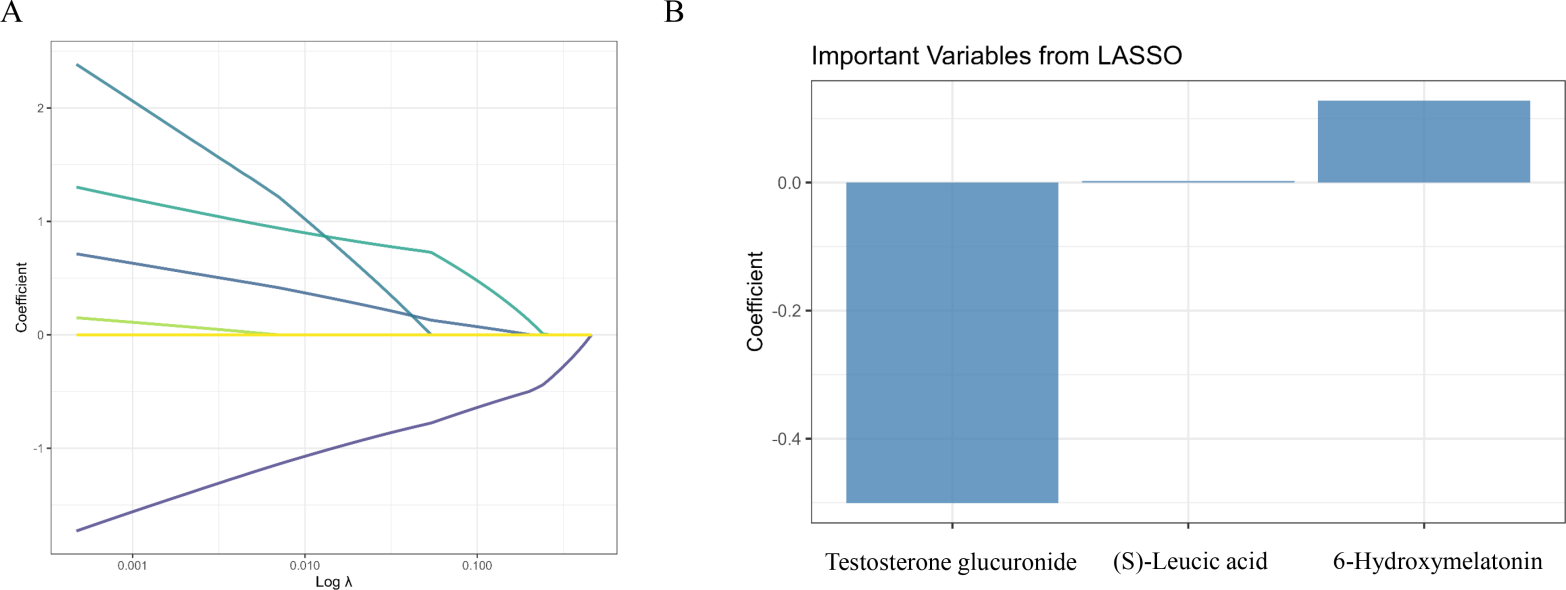
Screening of differential metabolites using LASSO regression. **(A)** LASSO regression cross-validation plot identifying the optimal λ to minimize deviance. **(B)** Bar plot of the regression coefficients for the three most important metabolites selected by the LASSO model.

**Figure 3** shows the results of LASSO regression and the subsequent analysis of selected metabolites. In **Figure 3A**, the coefficients of the metabolites are plotted against the log of the regularization parameter (λ), demonstrating the selection process for the most important metabolites in distinguishing between the control and PL group. **Figure 3B** shows the three most important metabolites selected by the LASSO model, namely, testosterone glucuronide, 6-hydroxymelatonin and (S)-leucic acid.

### Abundance and diagnostic performance of key metabolites

**Figure 4:**
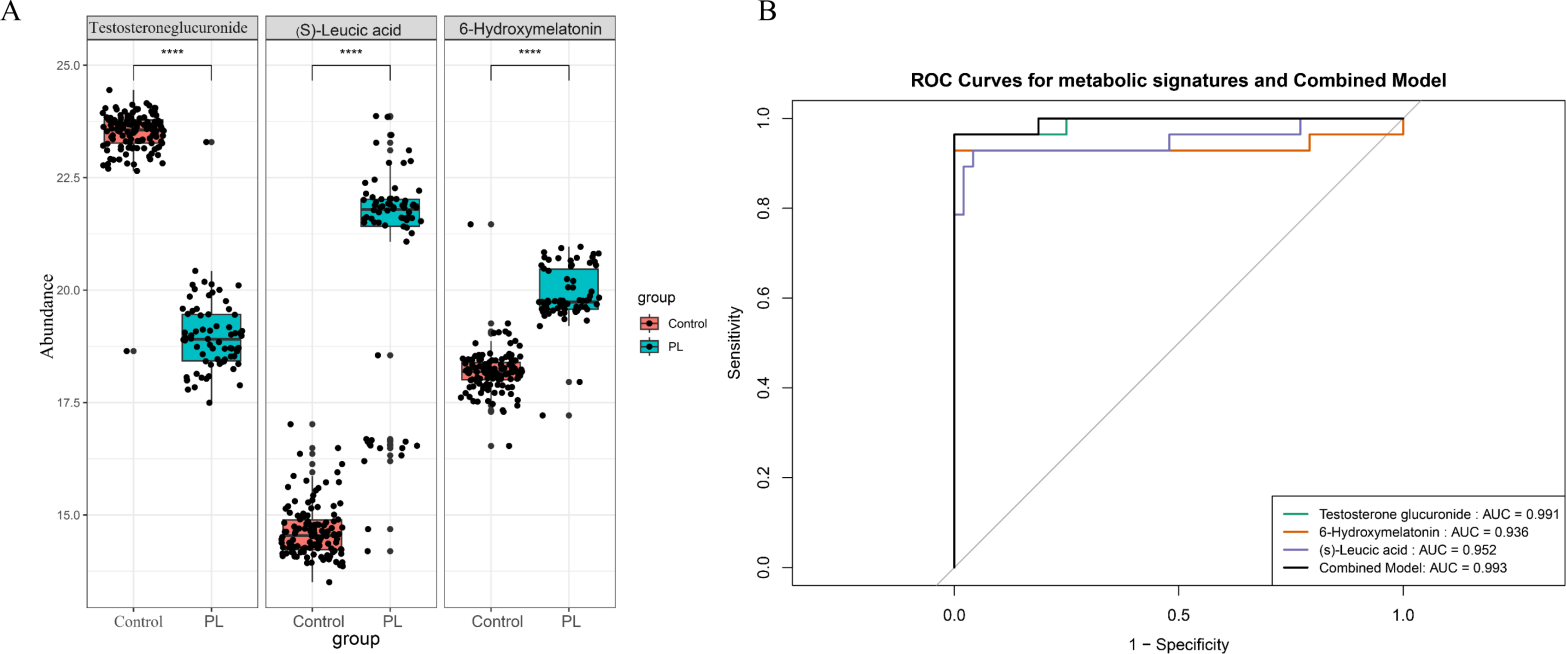
The most important metabolites and their diagnostic performance. **(A)** Box plots comparing the abundance levels of the three significant metabolites between the control and PL group (****p < 0.0001). **(B)** ROC curves demonstrating the diagnostic performance of a panel of metabolites in the test dataset.

**Figure 4A** compares the abundance levels of these metabolites between the control and PL group. Significant differences in abundance were observed for all three metabolites, with p values indicating significant differences (p<0.0001). These findings suggested that these metabolites play crucial roles in the metabolic alterations associated with PL. An ROC curve of the LASSO model revealed that the combined AUC value was 0.993, indicating high predictive accuracy for the selected metabolites in the validation set (**Figure 4B**). The performance of the optimal metabolite model was evaluated using a confusion matrix (**Supplementary Figure S1B**) and classification performance indicators (**Supplementary Figure S1C**). The confusion matrix revealed that the model achieved a sensitivity of 1.0000 and a specificity of 0.9643, indicating high diagnostic accuracy. In addition, the model had an accuracy of 0.9868. The positive predictive value (PPV) was 0.9796, and the negative predictive value (NPV) was 1.0000, reflecting the robust ability of the model to distinguish between the control and PL group.

### Comparative diagnostic performance of the key metabolites and clinical parameters

**Figure 5:**
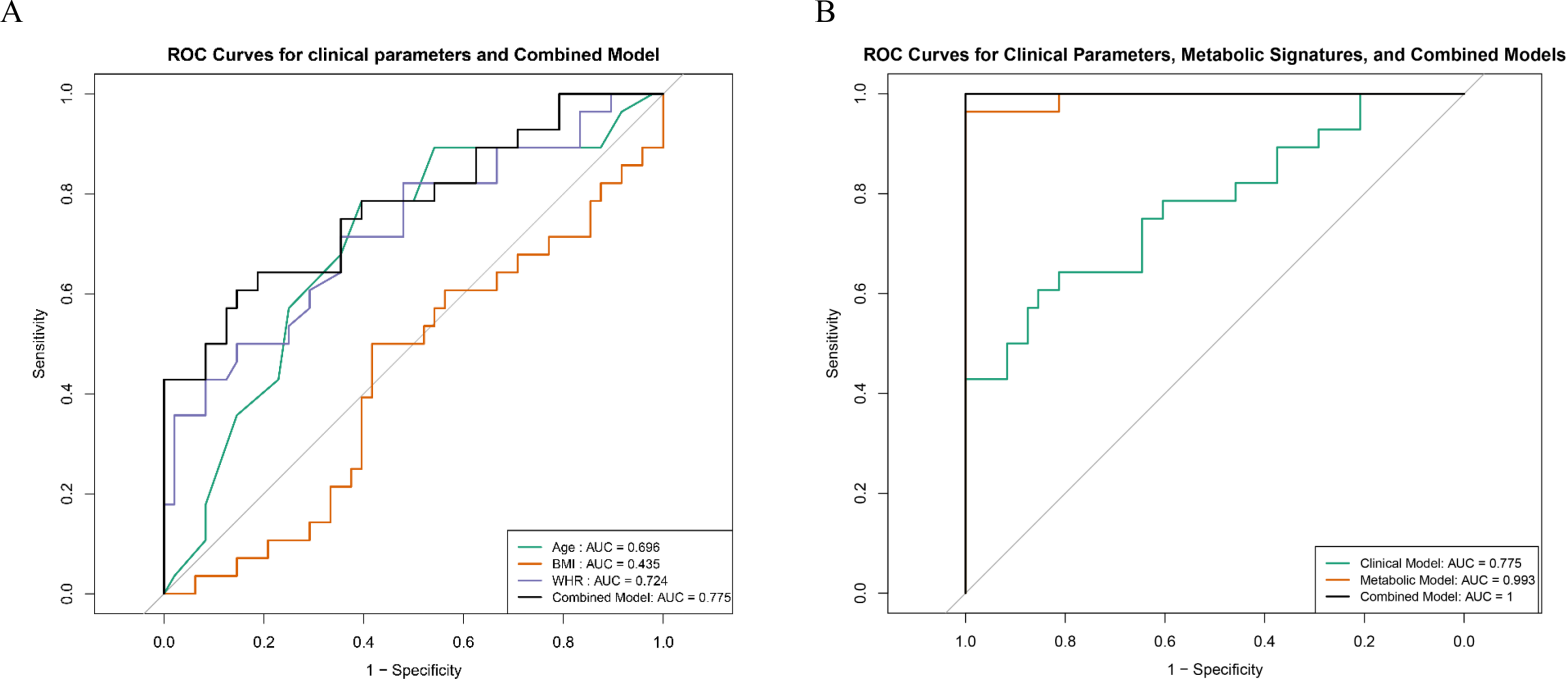
Comparative diagnostic performance of key metabolites and clinical indicators. **(A)** ROC curves illustrating the diagnostic performance of individual clinical parameters (age, BMI, and WHR). **(B)** ROC curves comparing the diagnostic performance of combined key metabolites, clinical parameters, and the integrated model.

The diagnostic performance of the clinical parameters and metabolites was evaluated via receiver operating characteristic (ROC) curve analysis. Among the clinical parameters, WHR had the highest AUC (0.724), followed by age (AUC = 0.696) and BMI (AUC = 0.435). The combined clinical model achieved an AUC value of 0.775 (**Figure 5A**). Furthermore, the combined model, which integrated both key metabolites and clinical parameters, achieved an AUC value of 1.000 (**Figure 5B**).

### Correlation analysis between differentially abundant metabolites and clinical parameters

**Figure 6:**
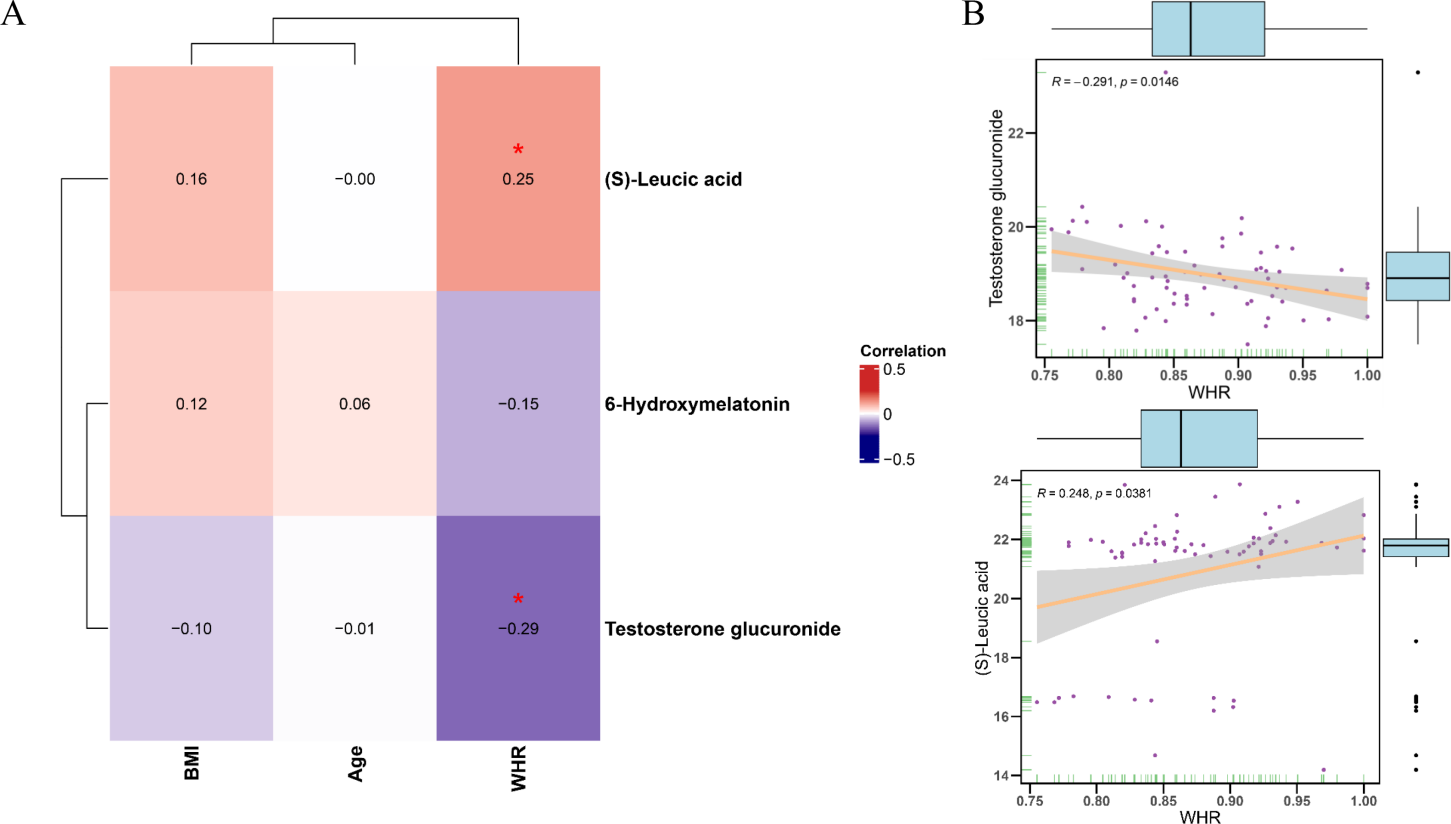
Correlation analysis between differential metabolites and clinical parameters in PL group. **(A)** Heatmap showing the Pearson correlation coefficients between core metabolites and clinical phenotypes parameters (age, BMI, WHR) in the PL group. **(B)** Scatter plots illustrating the significant correlations between WHR and testosterone glucuronide levels (negative correlation) and (S)-Leucic acid levels (positive correlation).

**Figure 6** shows the Pearson correlations between differentially abundant metabolites and clinical parameters within the PL group. The heatmap shown in **Figure 6A** depicts the Pearson correlation coefficients between core differentially abundant metabolites and clinical phenotypes. The scatter plot shown in **Figure 6B** further illustrates the negative correlation between WHR and testosterone glucuronide, which indicated that higher WHR values were associated with lower levels of testosterone glucuronide (r = -0.291, p = 0.0146), whereas (S)-leucic acid was significantly positively correlated with WHR (r = 0.248, p = 0.0381). Additionally, the correlation of WHR with the levels of testosterone glucuronide or (S)-leucic acid were evaluated in the control group. The scatter plot shown in **Supplementary Figure S1D** indicates that both correlations were nonsignificant in the control group (WHR vs. testosterone glucuronide: r = –0.0294, p = 0.748; WHR vs. (S)-leucic acid: r = 0.126, p = 0.168), suggesting no evident associations (**Supplementary Figures S1D, E**). Moreover, a permutation test comparing the correlation coefficients between the control and PL group revealed a statistically significant difference (p = 0.04) in the correlation between WHR and testosterone glucuronide, whereas the correlation between WHR and (S)-leucic acid did not differ significantly (p = 0.28).

## Discussion

In the present study, we identified differentially abundant metabolites between the control and PL group, which were primarily enriched in the caffeine metabolism, tryptophan metabolism, and riboflavin metabolism pathways. Caffeine metabolism emerged as a key pathway in the present analysis. Caffeine is widely consumed, and its metabolism involves several enzymes, such as cytochrome P450, which are integral to oxidative stress responses and liver function (25–27). Disruptions in caffeine metabolism may reflect dysregulated detoxification processes or oxidative stress, both of which are critical for maintaining pregnancy. Studies have suggested that high caffeine intake during pregnancy may increase the risk of pregnancy complications, including PL (28). Therefore, the significant alterations in caffeine metabolism observed in PL patients may reflect either direct caffeine exposure or dysregulated detoxification processes that impact pregnancy outcomes.

Amino acids play fundamental roles in various biological processes, including protein synthesis, immune regulation, and neuroendocrine function (29, 30). Our findings revealed significant alterations in amino acid metabolism pathways, with tryptophan metabolism emerging as a key pathway in PL patients. Tryptophan, an essential aromatic amino acid, plays a critical role in protein synthesis, immune regulation, and neuroendocrine function (31). The metabolism of tryptophan primarily proceeds through three major pathways, namely, the kynurenine (KYN) pathway, the serotonin (5-HT) pathway, and the indole pathway (32). The KYN pathway, which dominates tryptophan metabolism, is mediated by several enzymes, including tryptophan-2,3-dioxygenase (TDO) and indoleamine-2,3-dioxygenase (IDO). These enzymes are critical for modulating immune responses (33, 34). Dysregulated IDO activity has been associated with decreased kynurenine levels in PL patients, which may impair immune tolerance and increase the risk of fetal rejection. Additionally, tryptophan serves as a precursor for serotonin synthesis, a key neurotransmitter involved in regulating inflammation and maternal–fetal communication (35). Reduced tryptophan availability in PL patients may compromise serotonin production, exacerbating systemic inflammation and negatively affecting pregnancy outcomes (36). The present findings aligned with these observations, further supporting the critical role of tryptophan metabolism in pregnancy maintenance.

In addition, the present study revealed enrichment in the riboflavin metabolism pathway, highlighting its potential involvement in PL. Riboflavin, also known as vitamin B2, is a precursor for the synthesis of flavin adenine dinucleotide (FAD) and flavin mononucleotide (FMN), which act as essential cofactors for numerous redox enzymes involved in energy metabolism, oxidative stress regulation, and detoxification processes (37). Riboflavin metabolism plays a critical role in redox reactions, energy production, and cellular homeostasis (38, 39). Deficiency in riboflavin has been shown to impair mitochondrial function, resulting in disrupted ATP synthesis and increased production of reactive oxygen species (ROS) (40, 41). This imbalance may contribute to oxidative stress, a condition linked to adverse pregnancy outcomes, such as preeclampsia, intrauterine growth restriction, and spontaneous abortion (42, 43). In the present study, the significant alterations in riboflavin metabolism observed in PL patients may indicate disrupted mitochondrial energy production and heightened oxidative stress, both of which may compromise pregnancy maintenance.

LASSO algorithms were further employed to refine the selection of significant metabolites. The present analysis identified testosterone glucuronide, 6-hydroxymelatonin and (S)-leucic acid as key metabolites with strong diagnostic potential. These metabolites demonstrated high area under the curve (AUC) values in the ROC curve analysis, indicating their utility as noninvasive metabolites for PL and surpassing the diagnostic accuracy of clinical indicators alone. Testosterone glucuronide is a conjugated form of testosterone involved in androgen metabolism (44). In the context of PL, altered levels of testosterone glucuronide may reflect disruptions in hormonal regulation. 6-Hydroxymelatonin is a metabolite of melatonin, a hormone known for regulating sleep–wake cycles and reproductive functions (45). Changes in melatonin metabolism have been linked to various reproductive disorders, suggesting that alterations in melatonin metabolism in PL patients may indicate disruptions in circadian rhythms and reproductive health. (S)-Leucic acid is a metabolite of leucine, a branched-chain amino acid, and it has been studied for its anabolic effects (46), particularly in promoting muscle protein synthesis. However, the role of (S)-leucic acid in reproductive health and pregnancy remains largely unexplored. The present findings suggested that (S)-leucic acid may be involved in metabolic pathways that are critical for maintaining a healthy pregnancy.

Correlation analysis revealed a significant negative association between waist–to–hip ratio (WHR) and testosterone glucuronide in the PL group, implying that an elevated WHR may coincide with lower levels of testosterone glucuronide. Such disruptions could impair maternal–fetal communication and contribute to adverse pregnancy outcomes. Of note, this relationship was not observed in the control group, highlighting the specificity of metabolic alterations in PL patients. Although (S)-leucic acid was also positively correlated with WHR in the PL group, the difference in correlation coefficients compared with those in the control group was not statistically significant, possibly reflecting the limited sample size. Nevertheless, the apparent involvement of (S)-leucic acid in metabolic pathways relevant to pregnancy warrants further study to clarify its mechanistic role in PL and to assess its potential diagnostic value.

In conclusion, the present findings suggest that caffeine metabolism, tryptophan metabolism and riboflavin metabolism pathway may play important roles in the pathophysiology of PL, Moreover, testosterone glucuronide, 6-hydroxymelatonin, and (S)-leucic acid showed potential as noninvasive diagnostic metabolites. Further validation in larger cohorts is necessary to confirm these findings and to better understand the role of metabolic alterations in the pathophysiology of PL.

### Limitation

Despite the promising findings, this study has several limitations. Firstly, the sample size is relatively small, which may limit the generalizability of the results and prevent detailed stratification analyses, such as differentiating metabolic variations among PL cases with different recurrence histories. Secondly, the study design is cross-sectional, which prevents us from establishing causal relationships between the identified metabolic alterations and PL. Although we applied OPLS-DA and LASSO regression to prioritize key metabolites and minimize irrelevant variation and multicollinearity, these methods cannot fully account for confounding factors such as age, BMI, and WHR. We employed a permutation test to further address this issue, but future studies incorporating matched or statistically adjusted designs are warranted. Additionally, we did not collect detailed dietary information or account for variations in education level, which may influence metabolic profiles. Finally, the study focuses solely on plasma metabolites and includes limited clinical data. Future studies should aim to integrate multi-omics approaches and more comprehensive clinical datasets to provide a deeper understanding of the molecular mechanisms underlying PL.

## Data Availability

The original contributions presented in the study are included in the article/**Supplementary Material**. Further inquiries can be directed to the corresponding author.
