# Supplementary figures and images for: Metabolomics profiling identifies diagnostic metabolic signatures for pregnancy loss: a cross-sectional study from northwestern China
Source: Front Endocrinol (Lausanne). 2025 Apr 10;16:1518043. doi: 10.3389/fendo.2025.1518043 (PMC12018233; doi:10.3389/fendo.2025.1518043)

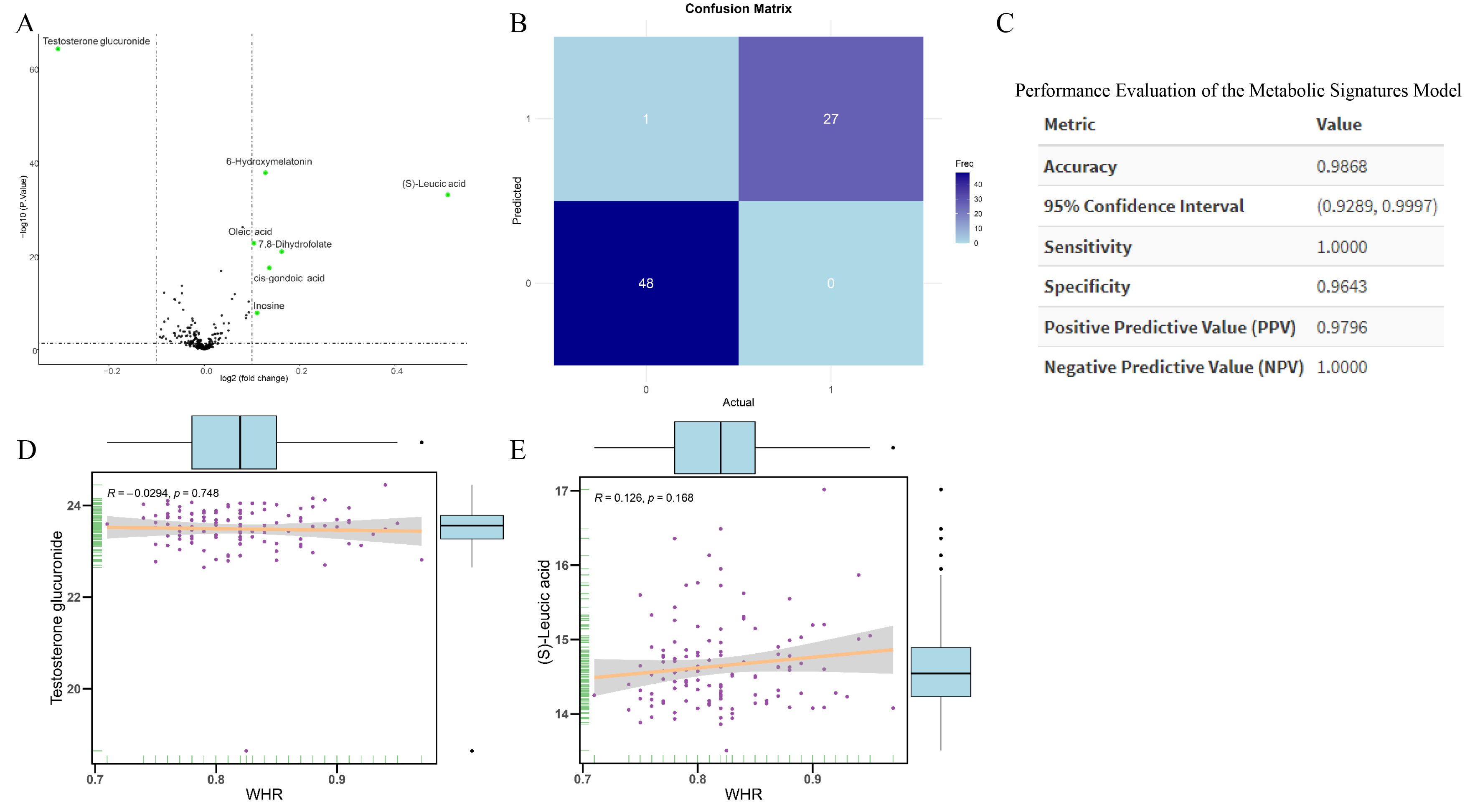

Supplement: Supplementary file 1 [file Image1.tif]
